# Primary Factors Influencing the Decision to Vaccinate against COVID-19 in the United States: A Pre-Vaccine Analysis

**DOI:** 10.3390/ijerph19031026

**Published:** 2022-01-18

**Authors:** Serkan Varol, Serkan Catma, Diana Reindl, Elizabeth Serieux

**Affiliations:** 1 Department of Engineering Management, University of Tennessee at Chattanooga, Chattanooga, TN 37403, USA; 2 Department of Business Administration, University of South Carolina Beaufort, Bluffton, SC 29902, USA; catma@uscb.edu; 3 Department of Nursing and Health Professions Business, University of South Carolina Beaufort, Bluffton, SC 29902, USA; dreindl@uscb.edu; 4 Institute for Health Metrics and Evaluation, University of Washington, Seattle, WA 98105, USA; eserieux@uw.edu

**Keywords:** vaccine hesitancy, COVID-19 vaccination, mask mandate, predictive modeling

## Abstract

Because vaccine hesitancy is a dynamic trait, it is critical to identify and compare the contributing factors at the different stages of a pandemic. The prediction of vaccine decision making and the interpretation of the analytical relationships among variables that encompass public perceptions and attitudes towards the COVID-19 pandemic have been extensively limited to the studies conducted after the administration of the first FDA-approved vaccine in December of 2020. In order to fill the gap in the literature, we used six predictive models and identified the most important factors, via Gini importance measures, that contribute to the prediction of COVID-19 vaccine acceptors and refusers using a nationwide survey that was administered in November 2020, before the widespread use of COVID-19 vaccines. Concerns about (re)contracting COVID-19 and opinions regarding mandatory face covering were identified as the most important predictors of vaccine decision making. By investigating the vaccine acceptors and refusers before the introduction of COVID-19 vaccines, we can help public health officials design and deliver individually tailored and dynamic vaccination programs that can increase the overall vaccine uptake.

## 1. Introduction

Since 11 March 2020, when the World Health Organization declared the novel coronavirus disease (COVID-19) outbreak a global pandemic, the world has grappled with how to contain and minimize its devastating effects [1]. To date, mandates to attempt to curtail the virus spread have varied from mask-wearing to curfews, to social distancing measures, and to other policy and behavioral interventions. Despite these measures, COVID-19, as of November 2021, was still one of the top causes of death in the US [2].

This virus has taken many lives globally and continues to cause immense suffering in every aspect of human life. The collaborative efforts of the scientific community, in conjunction with the support of governmental organizations, led to the development of various vaccines that are highly effective against severe disease caused by the original strain of COVID-19. For instance, after Israel vaccinated almost 60 percent of its population, as of April 23, 2021, the daily number of average fatalities dropped below 10, a steep decline from the average number of deaths (70) in January [3]. However, due to the introduction of new variants, such as Delta and Omicron, and a reduction in vaccine effectiveness over time, in August of 2021, Israel had an average of nearly 7500 daily confirmed cases [2]. The increase in cases led to an expansion of booster shots in Israel and globally.

The effectiveness of the available COVID-19 vaccines can be evaluated by comparing the mortality rates among vaccine recipients and unvaccinated individuals. For instance, the recipients of the booster shots who were 50 years of age or older due to the emergence of variants had 90% lower mortality than those who did not receive a booster in Israel during the summer of 2021 [4]. Similarly, according to the Vaccine Safety Datalink project initiated by the CDC’s Immunization Safety Office, the standardized mortality rate for recipients of two doses of the Pfizer-BioNTech vaccine was 0.37 person-years, significantly lower than that for unvaccinated people (1.11 person-years) between 14 December 2020 and 31 July 2021 in the US [5]. During the Delta surge in the summer of 2021, southern states such as Arkansas, Alabama, Mississippi, where older adults have the lowest vaccination rates, had the highest death rates from COVID-19 in the US [6].

Although progress is being made, and cases and deaths are diminishing primarily due to the vaccination campaigns being undertaken around the world and the addition of booster shots, vaccine hesitancy threatens to undermine or even halt this progress, especially as new variants of the virus continue to emerge. Knowing who would be likely to accept or reject the vaccine has critical importance, and could drastically change the trajectory of the pandemic across the globe. However, the trajectory of COVID-19 vaccine hesitancy shows that the indecision to be vaccinated has been a fluctuating process. More than one-third of vaccine-hesitant respondents before introducing COVID-19 vaccines leaned towards the willingness to be vaccinated after the actual vaccine administration process started in early 2021 [7]. If this dynamic nature of vaccine hesitancy is recognized by public health practitioners, health workers, and policy makers, individual messages and programs to target specific audiences at the different stages of the pandemic, in an attempt to diminish vaccine hesitancy, may result in a quicker end to the current and future pandemics. For any vaccination program to be successful, the highest vaccine uptake needs to be attained. However, vaccine hesitancy, the period of indecision about accepting a vaccine, continues to be a dynamic and complex concept that requires strategies that should be individually tailored based on the characteristics of the target populations [8].

The promising developments in the fight against this deadly virus are important milestones, but the pandemic is far from over. Addressing the global challenges, such as significant variation in governments’ capabilities, high vaccination costs, and the inability to effectively allocate and deploy vaccines [9], requires international coordination and collaborative efforts among developed and developing nations. Although it will take time and careful planning to end the pandemic worldwide, vaccinating at least 70–80% of the population to achieve herd immunity at a national level is not a distant dream for some countries, despite the continuous mutation of the virus. 

Much of the existing literature focuses on identifying the predictors that would impact the willingness to accept (WTA) vaccines using survey data. These variables range from the socio-demographic characteristics of the participants to the perceptions and attitudes towards the vaccines; hence, each variable’s statistical relationship with the WTA the vaccine can be investigated. However, although the results of these studies may have important implications for understanding vaccine hesitancy by offering an interpretation of the statistical relationships, the prediction of vaccine decision making [10,11] before the administration of the first FDA-approved vaccine has rarely been investigated. Thus, this study used predictive analytics to: (1) analyze the predictability of the vaccine acceptors and non-acceptors; and (2) identify the individual predictors that strongly influence the vaccine behaviors when COVID-19 vaccines were only hypothetical in nature. Investigating vaccine hesitancy around this baseline scenario is an important step to map out how willingness to be vaccinated evolved during the course of the pandemic. If vaccine hesitant groups, in addition to the predictors that impact their decisions, can be forecasted during the different times of the pandemic, individually tailored public health strategies may contribute to the success of current and future immunization programs by incorporating the dynamic nature of vaccine acceptance and refusal.

## 2. Materials and Methods

Based on a nationwide survey that was administered to capture the characteristics of the participants who expressed opinions about accepting or rejecting a COVID-19 vaccine, various predictive modeling techniques were performed in this study.

The first COVID-19 vaccine was administered in the US in December 2020. A month earlier, in November 2020, when the average number of daily cases was increasing after reaching the declines seen in September 2020, a nationwide survey was administered. Although the news about the potential vaccine approvals was circulating in the media, there was no FDA-approved vaccine during the administration of the survey. Thus, the results of this study can serve as baseline information.

A joint Institutional Review Board (IRB) application was filed and approved. QuestionPro (QuestionPro, Austin, TX, USA), an online survey software company, was contracted to gather the survey data using convenience sampling. A total of 1500 responses were collected regarding the participants’ socio-demographic backgrounds, health characteristics, and experiences with the virus, and whether they were willing to accept a COVID-19 vaccine. The socio-demographic characteristics of the participants are summarized in Table 1.

### 2.1. Data Preprocessing

Initially, anomalous data and outlier values were removed from the dataset. Then, the one-hot-encoding method was used to transform nominal categorical data into binary vectors. This resulted in a total of 1343 responses and 63 features that were used for the construction of our predictive models. Table 2 shows a set of individual-level indicators and the associated features.

The dataset, which consisted of 1343 cases, was split into a training set (80%) and a test set (20%). A 10-fold cross-validation method was used in the training sample for the performance evaluation of the classifier methods and hyperparameter tuning, discussed in the next section. A classifier may yield biased prediction accuracy when the dataset is imbalanced [12]. Therefore, the Synthetic Minority Over-Sampling Technique (SMOTE) was applied within each fold in the training set to address imbalanced data classification. SMOTE is an over-sampling technique that generates new synthetic minority classes by interpolating among neighboring minority class instances [13].

### 2.2. Machine Learning Classification

(1) Decision Tree (DT): A Decision Tree is a non-parametric supervised learning method that consists of several steps, including splitting (portioning data into subsets), pruning (reducing the size of the tree), and tree selection (finding the smallest tree that fits the data). It is often used in medical informatics and decision making in healthcare management [14]. Considering the size of our dataset and the number of variables, we chose to use the *rpart* algorithm in *R* that implements recursive partitioning, which allows for adjustable misclassification penalties.

(2) Random Forest Model (RFM): Random Forest classification techniques are composed of multiple decision trees which are frequently used in predicting events given their high-order interaction effects [15]. Each individual tree outputs a class prediction where the class having the most votes constitutes the model’s prediction. In RFM, the following parameters were adjusted to increase the model’s predictive power: (a) the number of trees to use, and (b) the minimum number of records allowed in a tree node.

(3) Logistic Regression Classifier (LR): Logistic regression is a probabilistic binary classifier that uses logit scores to predict the target class. It has been applied to many COVID-19 related studies, such as predicting infected patients’ recovery [16] and vaccinology [17]. In this research, a logit model was used to predict WTA a COVID-19 vaccination.

(4) Neural Network (NN): A Neural Network, an artificial intelligence method, is a subset of machine learning algorithms. The network consists of three layers, namely, an input layer, hidden layers, and an output player. It starts with an input layer which is fed by initial data (the features used for this study). The hidden layer is where all prediction computations are performed. The final phase, output layers, produces the results for the provided inputs. NN has been used in predicting vaccine utilization and targets in healthcare [18]. The hyperparameters in neural networks, such as the number of nodes in the hidden layers, the weight decay, the range of initial weights around zero, the maximum number of weights allowed in the model, and the maximum number of iterations for model estimation, are the design decisions that were optimized to maximize the performance of the model. 

(5) Naïve Bayes (NB): Naïve Bayes is a probabilistic predictive classifier that poses an assumption of independence among predictors, which helps in the accurate detection of classes. Naïve Bayes has higher accuracy when processing large patient data points [19]. In this study, we used the Laplace smoothing technique to smooth categorical data. 

(6) Support Vector Machine (SVM): The Support Vector Machine is a versatile machine learning technique that is frequently adopted for the classification and segregation of patients’ clinical data in the healthcare sector [20]. SVM performs the classification process by finding the hyperplane that optimally separates the data. In order to find the most optimal hyperplane that separates two classes of instances, we used the grid search method as an approach to hyperparameter tuning for the popular Linear, Polynomial, Radial, and Sigmoid kernels. 

### 2.3. Evaluation and Comparison of the Models

A confusion matrix (Table 3) was created to analyze the performance of each selected modeling technique, which specifically measures how well the models fit predicting prospective (validation data set) outcomes using true positive (TP), true negative (TN), false positive (FP), and false negative (FN) values. A true positive is an outcome where the model correctly predicted the individuals who intended to get vaccinated. Similarly, a true negative is an outcome where the model correctly predicted individuals who did not intend to get vaccinated. Both false positive and false negative metrics indicate incorrect predictions of given classes.

Accuracy, precision, sensitivity, and specificity metrics are defined in terms of TP, TN, FP, and FN. Accuracy presents the number of correct predictions in the model. Precision is the ratio between the true positive and all the positive instances. Sensitivity is the rate of true positives, and specificity is defined as the ratio of true negatives.

In the presence of the data balance distribution, the accuracy, sensitivity, and specificity measures were chosen to assess the performance of each selected model and class. We also report the F1 value, which provides the weighted mean of precision and sensitivity, which takes false positives and false negatives into account. In addition, the area under the receiver operating characteristics curve (AUC-ROC) was used to measure and compare the performance of the classification models at different probability thresholds.

## 3. Results

A comparison of the selected models is shown in Table 4. The SVM model led to an improvement in predictive performance with a 77.40% accuracy rate, where a much higher accuracy rate was obtained when predicting vaccine acceptors (sensitivity) as compared to vaccine refusers (specificity). Moreover, the model reported the highest F1 score (76.12%). A higher F1 score indicates a better performing model; thus, a lower number of false positives and false negatives are predicted via the model. Simply stated, the model does a better job when assessing false negatives (individuals who were predicted to be “vaccine refusers” but actually were “vaccine acceptors”) and false positives (individuals who were predicted to be “vaccine acceptors” but actually were “vaccine refusers).

**Figure 1:**
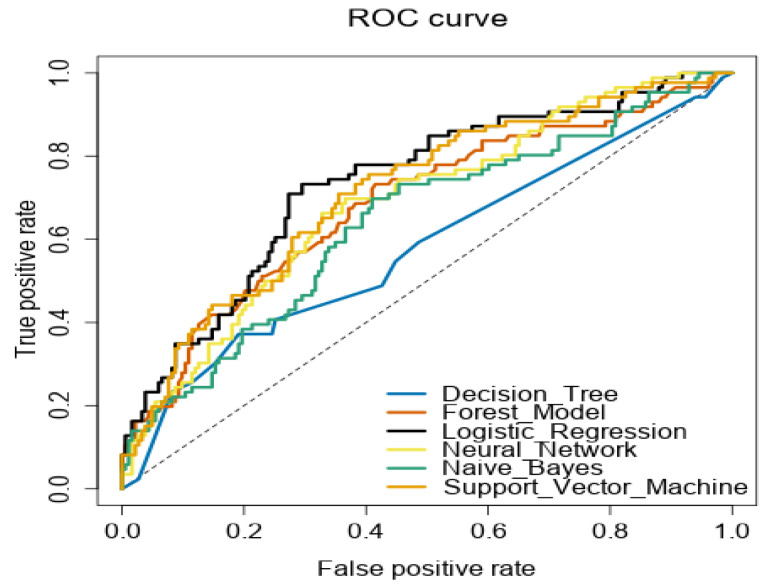
The receiver operating characteristic (ROC) curve shows the performance of each predictive model at different classification thresholds.

An ROC curve was also used to compare the classification performance of the selected predictive models. A curve closest to the top left corner in an ROC plot indicates the space where the highest TP and lowest FP rates are detected. The area under the ROC curve is a measure of the model’s discriminative abilities (e.g., how well a model can distinguish between vaccine acceptors and vaccine refusers). Figure 1 presents the ROC curves of the selected models. The value of the area under the curve (AUC) was computed and is shown in Table 4. Logistic regression was found to attain the highest AUC score (74.15%), and has the best measure of separability in comparison to the other models (i.e., logistic regression can be used in the case of observing the tradeoff between true positive rate and false positive rate).

## Feature Importance

A variable importance analysis was performed to assess the characteristics that contribute to the overall predictions of vaccine acceptors and refusers. Variable importance was computed using the mean decrease in the Gini coefficient, which measures the mean decrease in node impurity [21]. Higher importance of the prediction is indicated by a higher value of the mean decrease in the Gini coefficient.

**Figure 2:**
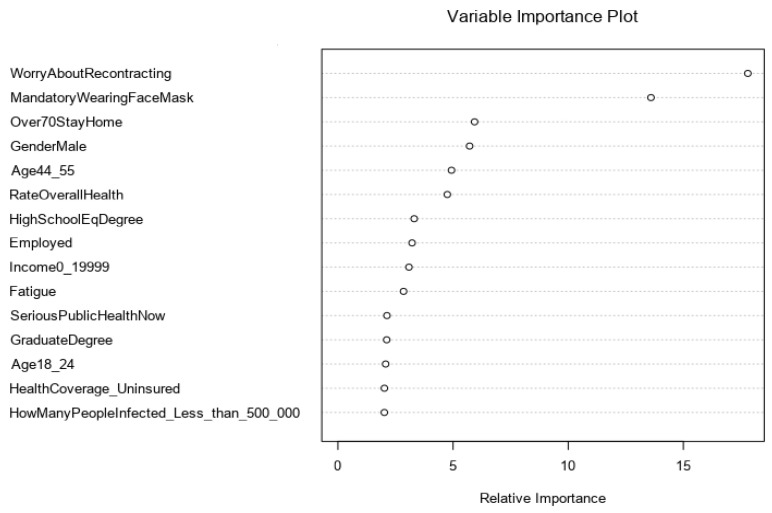
The variable importance plot shows the most significant predictors of vaccine acceptors and refusers. The top variables with higher values of the mean decrease in the Gini coefficient contribute more to the model.

Figure 2 ranks the top 15 features and reports the most relevant and important factors when classifying both vaccine acceptors and refusers. Worrying about (re)contracting COVID-19 was the leading predictive feature, followed by opinions regarding mandatory face covering. These were found to be more important than the other policy measures, such as closing gyms, restaurants, and shops, mandatory self-quarantine, and curfews, when identifying classes. In addition, the perceived seriousness and the threat of the pandemic was a strong indicator for distinguishing between vaccine acceptors and refusers.

Various socioeconomic factors, such as gender, employment status, and income level, have a significant impact on the outcome values. Gender has the most predictive power among other socioeconomic factors. The group of individuals aged between 44 and 55 years has more discriminative power than any other age group. Moreover, the income range of $0 to $19,999 is ranked as the most effective income-related factor of the vaccination intent. It is important to note that marital status held the least explanatory value among all other socioeconomic-related variables.

## 4. Discussion

This study aimed to (1) analyze the predictability of the vaccine acceptors and non-acceptors, and (2) establish a hierarchy of features impacting the decision to be vaccinated against SARS-CoV-2. The results offer insights into the willingness to accept or reject the COVID-19 vaccine before any of the current COVID-19 vaccines were approved and administered. This issue has been rarely studied in the literature.

In pursuit of the first objective, we compared six different machine learning models to examine the decision to vaccinate. This study found that the Support Vector Machine has the highest predictive performance accuracy rate (77.40%). This simply means that the model correctly classifies 77.4% of the vaccine acceptors and refusers. However, the higher sensitivity score indicated that SVM performed better when predicting vaccine acceptors as compared to vaccine refusers. The ability to predict vaccine acceptors in a community is tremendously helpful for many reasons, including the fact that it can, when considered as a percentage of the overall community population, also provide an indication of the level of hesitancy in that same community. The “3 Cs” model of vaccine hesitancy highlights that the determinants of hesitancy fall under three main categories: complacency, convenience, and confidence [22]. Therefore, although more work is needed to determine the specific causes of vaccine hesitancy in a population, public health messages can reduce complacency, emphasize convenience, and increase convenience. The model yielded an F1 score of 0.7612 (76.12%), which is indicative of a better performing model, and thus produces a lower number of false positives and false negatives. This means that this model does a better job when assessing individuals who were predicted to be “vaccine refusers” but actually were “vaccine acceptors” (false negatives) and individuals who were predicted to be “vaccine acceptors” but actually were “vaccine refusers” (false positives). Taken together, therefore, these models may be helpful in predicting the levels of vaccine hesitancy in specific communities, thereby enabling the development and implementation of tailored programs aimed at increasing vaccine uptake in areas where it would have taken longer or not happened at all. This would confer a huge advantage in dealing with this current COVID-19 pandemic and future outbreaks.

Objective two sought to determine the individual predictors that strongly influence vaccine behaviors. The two strongest predictors were (a) worrying about (re)contracting COVID-19 and (b) considerations regarding masking or face coverings. This finding is significant as it provides a foundation on which to build and implement campaigns to promote vaccine acceptance. Our findings suggest that the CDC guidelines from May 2021, in which fully vaccinated people could resume activities without wearing masks except in specific cases, may actually increase vaccine acceptance and result in more people becoming vaccinated. One of the main tenets of operant conditioning is that behavioral responses are primarily influenced by experiences with reinforcement; negative reinforcement refers to influencing behavior through the removal of something aversive. Simply put, in this context, if the consequence of a behavior is something aversive (e.g., being unvaccinated requires mask-wearing) then that behavior, in this case, vaccine hesitancy, is likely to be reduced when faced with the possibility of mask use discontinuation. Therefore, our study indicates that being able to stop wearing masks after vaccination may be one of the most effective strategies to promote vaccine acceptance. Our study also found that worrying about (re)contracting COVID-19 was the other strongest individual predictor for vaccine behavior. This is significant in our current context because, although we have not yet determined the length of time for which vaccines offer protection, we know that they protect against severe illness and reduce transmission. Our study indicates that public health messaging should focus on these two aspects to have the greatest impact on promoting vaccine acceptance, especially during the early stages of a pandemic, when vaccines are not readily available. Further, once the period of vaccine conferred immunity has been established, and the associated necessity of booster shots, widespread dissemination of this information may promote vaccine acceptance because this directly addresses the possibility of reinfection and would reduce concerns about (re)infection.

The identification of two prominent features influencing WTA has practical implications for vaccination programs across the United States. If the only two messages used to influence WTA a vaccine focused on reducing the worry about (re)contracting COVID-19 and avoiding the need for mandatory face masks, a vast majority of individuals would choose to be vaccinated in the United States. These two features are identified as the most prominent in influencing WTA a COVID-19 vaccine. Vaccination program messaging across the United States warrants attention regarding the two most predictive features contributing to the decision to accept a COVID-19 vaccine, i.e., reducing the worry about (re)contracting COVID-19 and avoiding the need for mandatory face masks. However, it should be noted that these predictors may have varying degrees of importance depending on the trajectory of pandemic.

Our additional findings that gender, age, education, and income level have a significant impact on vaccine behavior may also be valuable in creating strategies to promote vaccine acceptance. Specifically, strategies segmenting the population and focusing on gender, the age group 44–55, the income range of $0 to $19,999, and attainment of High School (or equivalent) degrees may be more effective at converting hesitancy to acceptance than more broadly focused population-wide strategies. Moreover, the perceived seriousness and threat of the pandemic was a strong indicator for distinguishing between vaccine acceptors and refusers, which supports the main premise of the Health Belief Model. This states that individual beliefs regarding susceptibility and severity of a health concern, in addition to the beliefs about the effectiveness of possible preventive action, predict the likelihood of behavior [23]. This is, people will take action if they think they are susceptible to a condition which will have serious consequences for them, if they also believe that the action will be beneficial to them. Thus, taken with our findings, this suggests that messages and campaigns focusing on susceptibility, severity, and vaccine efficacy may also increase vaccine acceptance.

## 5. Conclusions

Our results suggest that messages focused on (re)contracting COVID-19 would be most effective in promoting vaccine acceptance, and messages addressing the possibility of no longer wearing face masks once vaccinated would play a significant role in increasing vaccine acceptance before the approval of vaccines. However, a detailed analysis of vaccine acceptance and its determinants requires the acknowledgment that the decision or indecision to be vaccinated may not remain constant during the period of pandemic. Although the results presented in this study may serve as a baseline of vaccine hesitancy, the findings of similar studies that looked at this important issue after the widespread use of initial vaccines, and after booster shots, will help us understand how vaccine hesitancy evolved during the course of the pandemic. Only then will public health officials be able to devise optimal programs that will be aimed at increasing vaccine acceptance.

This study was subject to limitations. Due to the lack of quota sampling, the data collected may not be the representative of the US population. Secondly, because the personal information needed for follow-up information was not obtained during the data collection, a follow-up study, after the administration of the initial vaccines and booster shots, was not conducted. Given the cross-sectional nature of the design, the link between cause and effect cannot be established, and all possible relevant survey responses may not have been included.

Future research would benefit from a comparison of the features contributing to the decision to reject the COVID-19 vaccine before and after the introduction of COVID-19 vaccines. To predict the willingness to accept or reject the COVID-19 vaccine during the different stages of pandemics is a valuable tool for public health professionals and efforts to vaccinate the United States population. Other features to be considered in future research exploring the core of vaccine rejection include mandates, geography, religion, and spirituality. Examining the degree to which individuals practice preventative measures post-vaccination may warrant examination.

## Figures and Tables

**Table 1:** Socio-demographic characteristics of the participants (N = 1343).

*Variable*	*Definition*	*N (%)*
*Age*	*Age of the respondent:*	
	1 if between 18 and 24	179 (13%)
	2 if between 25 and 35	312 (23%)
	3 if between 36 and 45	227 (17%)
	4 if between 46 and 55	188 (14%)
	5 if between 56 and 65	197 (15%)
	6 if more than 65	240 (18%)
Gender	1 if respondent is male, 0 = female	415 (31%)
Ethnicity	1 if respondent is White, 0 = otherwise	996 (74%)
Marital Status	1 if respondent is married, 0 = otherwise	601 (45%)
Education	Highest level of education of the respondent:	
	Less than high school	33 (2%)
	High school	260 (19%)
	Some college	316 (24%)
	Associate degree	197 (15%)
	Bachelor’s degree	362 (27%)
	Graduate degree	175 (13%)
Income	Annual family income:	
	1 if <$20,000	280 (21%)
	2 if between $20,000 and $39,999	295 (22%)
	3 if between $40,000 and $59,999	266 (20%)
	4 if between $60,000 and $79,999	186 (14%)
	5 if between $80,000 and $99,999	102 (8%)
	6 if more than $100,000	214 (16%)
Employment Status	1 if respondent is employed, 0 = otherwise	1032 (77%)
Health Care Worker	1 if respondent is a health-care worker, 0 = otherwise	338 (25%)

**Table 2:** Definitions of features used in the study.

*Variable*	*Levels*
***Socio-Demographic***	
Age	[(18–24), (25–35), (36–45), (46–55), (56–65), (>65)]
Gender	1 = Male, 0 = Female
Ethnicity	1 = White, 0 = Others
Marital status	1 = Married, 0 = Others
Education	Less than high school, High school diploma, Some college education, Associate degree, Bachelor’s degree, Graduate degree (Master’s or Doctorate)
Income	Less than $20,000, $20,000–$39,999, $40,000–$50,999, $60,000–$79,999; $80,000–$99,999, Equal to or more than $100,000
Employment status	1 = Employed, 0 = Not employed
Healthcare worker	1 = Healthcare worker, 0=Not healthcare worker
***Health Background***	
Health insurance coverage	Affordable Care Act, Medicaid, Medicare, Private health insurance, Uninsured, Other health coverage.
Self-rated overall health of the participant	Excellent, Very good, Good, Fair, Poor
Living with anyone with at least one pre-existing condition	1 = Yes, 0 = No
Respondent was tested positive for COVID-19	1 = Yes, 0 = No
Respondent was hospitalized for COVID-19	1 = Yes, 0 = No
Respondent was worried about re-contracting the virus	1 = Yes, 0 = No
Living with anyone who was tested positive for COVID-19	1 = Yes, 0 = No
Family member died because of COVID-19	1 = Yes, 0 = No
***Awareness and Knowledge of COVID-19***	
How many people do you think have been infected with COVID-19 in the US?	Less than 500,000, 500,001–1,000,000, 1,000,001–3,000,000, 3,000,001–5,000,000, More than 5,000,000, I do not know
Which of the following do you think are the symptoms of COVID-19 (select all that apply)?	Fever or chills, Cough, Shortness of breath or difficulty breathing, Fatigue, Muscle or body aches, Headache, New loss of taste or smell, Sore throat, Congestion or runny nose, Nausea or vomiting, Diarrhea, I do not know
What measures do you think should be taken to prevent the spread of COVID-19 virus?	Wash hands with water and soap for 20 s, Avoid touching the eyes, nose and mouth with unwashed hands, Avoid close contacts with infected people, Covering mouth and nose when coughing or sneezing, Covering mouth and nose with a mask when around others, Avoid shaking hands, Clean and disinfect frequently touched surfaces daily, Closing windows at home, Wearing gloves all times, I do not know
What are the ways through which COVID-19 Virus is contracted?	Close contact (within 6 feet) with an infected person who has symptoms, Close contact (within 6 feet) with an infected person even if they aren’t showing symptoms of infection, Contact with surfaces an infected person has touched, I do not know
***Perceptions and Attitude towards COVID-19***	
How serious of a public health threat did you think the coronavirus was when you first heard about it?	Not serious at all, Not too serious, Somewhat serious, Serious, Very serious
How serious of a public health threat do you think the coronavirus is now?	Not serious at all, Not too serious, Somewhat serious, Serious, Very serious
How would you rate the federal government’s efforts to control the COVID-19 Pandemic?	Not effective at all, Hardly effective, Somewhat effective, Effective, Very effective
***Please state the effectiveness of the following policy measures:***	
Close schools and daycares	Not effective at all, Hardly effective, Somewhat effective, Effective, Very effective
Close gyms/restaurants	Not effective at all, Hardly effective, Somewhat effective, Effective, Very effective
Close all shops except for supermarkets and pharmacies	Not effective at all, Hardly effective, Somewhat effective, Effective, Very effective
Don’t allow visitors in hospitals, nursing homes, and elderly homes	Not effective at all, Hardly effective, Somewhat effective, Effective, Very effective
Oblige people aged 70 and over or with a medical condition to stay at home except to do basic shopping or because urgent medical attention is required	Not effective at all, Hardly effective, Somewhat effective, Effective, Very effective
Oblige everyone who does not work in a crucial professional group (for example, people who work in healthcare, public transport, the food chain) to stay at home except to do basic shopping or because urgent medical care is required	Not effective at all, Hardly effective, Somewhat effective, Effective, Very effective
Mandatory wearing of face masks	Not effective at all, Hardly effective, Somewhat effective, Effective, Very effective
Mandatory self-quarantine for travelers from a state with high infection rate	Not effective at all, Hardly effective, Somewhat effective, Effective, Very effective
Restrict international travel	Not effective at all, Hardly effective, Somewhat effective, Effective, Very effective

**Table 3:** Confusion matrix.

Metric Name	Formulas for Confusion Matrix
Accuracy	$\frac{TP+TN}{TP+TN+FP+FN}$
Precision	$\frac{TP}{TP+FP}$
Sensitivity	$\frac{TP}{TP+FN}$
Specificity	$\frac{TN}{TN+FP}$

**Table 4:** A comparison of selected predictive models (fit and error measures).

Model	Accuracy	F1	AUC	Specificity	Sensitivity
Decision Tree	0.6681	0.6637	0.5817	0.6573	0.6807
Forest Model	0.7662	0.7538	0.6910	0.7170	0.8170
Logistic Regression	0.7543	0.7380	0.7415	0.6981	0.8138
Neural Network	0.7134	0.7109	0.6998	0.7067	0.7197
Naïve Bayes	0.7338	0.7178	0.6671	0.6798	0.7883
Support Vector Machine	0.7740	0.7612	0.7199	0.7230	0.8260

## Data Availability

The data presented are available on request from the corresponding author.
